# Association between urinary polycyclic aromatic hydrocarbon metabolites and premature menopause: a nationally representative cross-sectional study in the United States

**DOI:** 10.1265/ehpm.25-00031

**Published:** 2025-05-02

**Authors:** Qian Yang, Lingling Zeng, Jinfa Huang, Jianxiong Wuliu, Hai Liang, Kaixian Deng

**Affiliations:** Department of Gynecology, Shunde Hospital, Southern Medical University (The First People’s Hospital of Shunde, Foshan), Foshan 528308, PR China

**Keywords:** Premature menopause, Polycyclic aromatic hydrocarbons, Ovarian function, NHANES, Cross-sectional study

## Abstract

**Background:**

Premature menopause, defined as natural menopause before age 40, is associated with diminished ovarian reserve. Despite growing concerns regarding environmental pollutants, no large-scale population-based studies have systematically examined the association between urinary polycyclic aromatic hydrocarbon metabolites (UPAHMs) and premature menopause.

**Methods:**

This cross-sectional study analyzed 2001–2020 NHANES data, including urinary levels of six PAH metabolites: 1-naphthol (1-NAP), 2-naphthol (2-NAP), 3-fluorene (3-FLU), 2-fluorene (2-FLU), 1-phenanthrene (1-PHE), and 1-pyrene (1-PYR). Premature menopause was self-reported as natural menopause occurring before age 40. Multivariable logistic regression assessed UPAHMs’ association with premature menopause, with restricted cubic splines (RCS) evaluating nonlinear trends. Subgroup analyses examined demographic interactions.

**Results:**

Among 2,565 participants, 662 reported premature menopause. Multivariable logistic regression showed significant associations between elevated urinary levels of 1-NAP (OR: 1.01, 95% CI: 1.00–1.02, P = 0.02), 2-NAP (OR: 1.01, 95% CI: 1.00–1.02, P = 0.02), and 3-FLU (OR: 1.03, 95% CI: 1.01–1.05, P = 0.01) and increased risk of premature menopause. RCS analysis revealed significant nonlinear relationships for 2-NAP, 3-FLU, 2-FLU, 1-PHE, and 1-PYR with premature menopause risk. White participants showed greater susceptibility to UPAHMs.

**Conclusion:**

Elevated UPAHMs, particularly 1-NAP, 2-NAP, and 3-FLU, were linked to higher premature menopause risk, with nonlinear trends observed. White individuals demonstrated greater vulnerability, emphasizing the need for targeted interventions to reduce PAH exposure.

## Introduction

Premature menopause, or premature ovarian insufficiency (POI), is defined as the cessation of menstruation before age 40 due to irreversible ovarian functional decline, affecting approximately 1% of women [1–3]. Beyond fertility loss, it is linked to heightened risks of cardiovascular disease, osteoporosis, cognitive decline, depression, and increased all-cause mortality [4, 5]. This condition accelerates systemic aging and significantly impacts women’s mental health and quality of life. While its etiology is multifactorial, involving genetics, autoimmunity, and lifestyle factors, environmental pollutants have emerged as key contributors to ovarian dysfunction [6, 7].

Polycyclic aromatic hydrocarbons (PAHs) are persistent organic pollutants widely present in the environment, originating from sources such as fossil fuel combustion, industrial emissions, cooking fumes, and cigarette smoke [8, 9]. In the United States, the Environmental Protection Agency (EPA) has designated 16 PAHs as priority pollutants due to their potential health risks [10]. PAH emissions are regulated under environmental policies such as the Clean Air Act, which sets air quality standards for industrial emissions and vehicular pollutants [11]. However, despite these regulatory efforts, environmental PAH exposure remains widespread due to ongoing emissions and incomplete mitigation strategies. Once absorbed into the body, PAHs are metabolized by the cytochrome P450 enzyme system into bioactive metabolites, such as epoxides and dihydrodiols, which impair ovarian function through multiple pathways [12, 13]. These metabolites induce oxidative stress, DNA damage, and follicular atresia in ovarian granulosa cells and oocytes, reducing follicular reserves [14–16]. They also trigger local ovarian inflammation and apoptotic signaling, further compromising ovarian structure and function [17]. Although experimental studies have linked PAH exposure to ovarian decline, large-scale epidemiological evidence connecting PAHs with premature menopause risk is limited.

To better evaluate the impact of environmental PAH exposure on reproductive health and optimize regulatory strategies, this study utilized data from the 2001–2020 National Health and Nutrition Examination Survey (NHANES) to assess the association between UPAHMs concentrations and the risk of premature menopause. By identifying high-risk populations, this study aims to elucidate the potential effects of PAH exposure on reproductive health and provide scientific evidence to support PAH regulation, public health policymaking, and targeted interventions for vulnerable populations.

## Methods

### Data source and study design

This study was based on cross-sectional data from the 2001–2020 NHANES. NHANES is a nationally representative, continuous survey designed to assess the health and nutritional status of the U.S. population [18]. Data were collected through standardized questionnaires, physical examinations, and laboratory tests.

### Study population

**Fig. 1:**
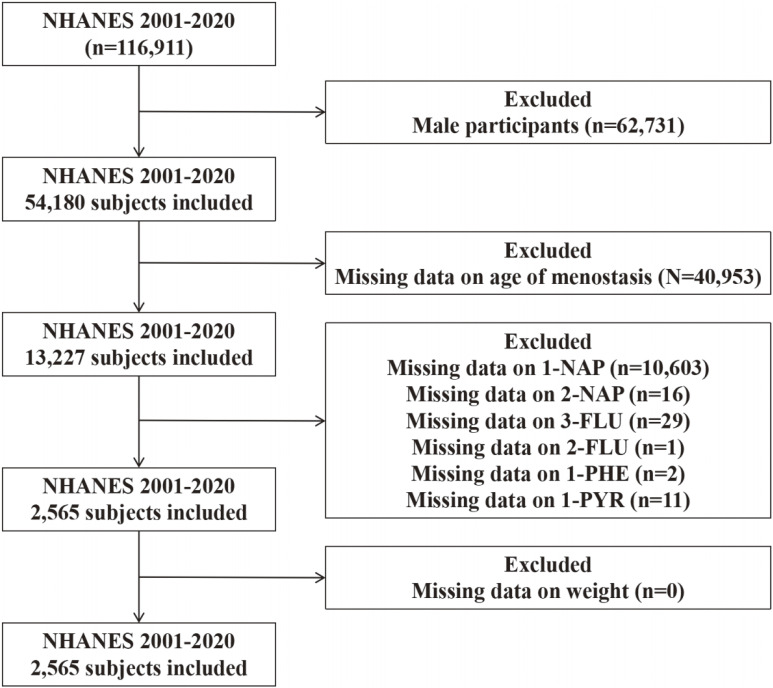
Flowchart of participant selection for the study on UPAHMs and premature menopause.

A total of 116,911 participants from the 2001–2020 NHANES dataset were initially included. After excluding male participants (n = 62,731), 54,180 female participants remained. Participants with missing data on menopausal age (n = 40,953) were further excluded, leaving 13,227 participants. Among these, individuals with missing UPAHMs data for 1-NAP (n = 10,603), 2-NAP (n = 16), 3-FLU (n = 29), 2-FLU (n = 1), 1-PHE (n = 2), and 1-PYR (n = 11) were excluded. Participants without weight data were excluded (n = 0). The final analytic sample consisted of 2,565 participants (Fig. 1).

### Assessment of premature menopause

Premature menopause was assessed using NHANES reproductive health questionnaire data [19–21]. First, participants who reported not having menstruated in the past 12 months (rhq031) were identified. Among them, only participants who attributed this to menopause (rhq040) were retained. The age of menopause was determined based on the response to “How old were you when you had your last menstrual period?” (rhq060). Participants who reported natural menopause before age 40 were classified as the premature menopause group, while those who reported menopause at age 40 or later were categorized as the control group.

### UPAHMs assessment

Urine samples were collected in sterile containers during NHANES examinations, frozen at −20 °C, and transported to CDC laboratories for analysis. Six UPAHMs—1-hydroxynaphthalene (1-NAP), 2-hydroxynaphthalene (2-NAP), 3-hydroxyfluorene (3-FLU), 2-hydroxyfluorene (2-FLU), 1-hydroxyphenanthrene (1-PHE), and 1-hydroxypyrene (1-PYR)—were quantified using high-performance liquid chromatography-tandem mass spectrometry (HPLC-MS/MS). Metabolites were enzymatically hydrolyzed using β-glucuronidase/arylsulfatase, followed by solid-phase extraction and quantification. Results were reported in nanograms per milliliter (ng/mL) and normalized for urinary creatinine concentration (µg/g creatinine) to account for dilution differences [22, 23]. Detailed procedures are available in the NHANES Laboratory Procedure Manual (https://www.cdc.gov/nchs/nhanes/).

### Covariates

Covariates included body mass index (BMI), race (Mexican American, non-Hispanic Black, non-Hispanic White, other), education (less than high school, high school, more than high school), poverty-to-income ratio (PIR, continuous) [24, 25], smoking status (yes/no), alcohol consumption (yes/no), hypertension (yes/no), diabetes (yes/no), marital status (separated, married, never married), pregnancy (yes/no), and urinary creatinine. Covariates were obtained through NHANES standardized questionnaires, physical examinations, and laboratory tests. Demographic and lifestyle variables were collected via structured interviews, while clinical variables were based on self-reported medical history and examination findings. Hypertension and diabetes were self-reported by participants [26, 27].

### Statistical analysis

All analyses were performed using R software (version 4.2.2), with appropriate NHANES sampling weights to ensure national representativeness. Multiple imputation addressed missing data to reduce bias. Continuous variables were summarized as weighted means with standard errors (SE), and categorical variables as weighted percentages. Multivariable logistic regression evaluated the associations between UPAHMs and premature menopause, reporting odds ratios (OR) and 95% confidence intervals (CI). Models adjusted for BMI, education, PIR, age, race, smoking, alcohol consumption, hypertension, diabetes, marital status, pregnancy history, and urinary creatinine. Restricted cubic spline (RCS) analyses assessed nonlinear relationships, with overall and nonlinearity P values reported. Subgroup analyses stratified participants by demographic and clinical factors, testing for interaction significance to identify effect modifiers. All statistical tests were two-sided, and a significance level of P < 0.05 was considered statistically significant.

## Results

### Population characteristics

Baseline characteristics stratified by menopausal status are shown in Table 1. Compared to the normal menopause group, participants in the premature menopause group were younger (P < 0.001) and had lower PIR (P < 0.001), while BMI showed no significant difference. Urinary levels of 2-NAP, 3-FLU, 2-FLU, 1-PHE, 1-PYR, and urinary creatinine were significantly higher in the premature menopause group (P < 0.05), but 1-NAP levels showed no difference. The premature menopause group included higher proportions of Mexican American and Black participants and fewer White participants (P = 0.03). They were also less likely to have education beyond high school (P = 0.001), had lower hypertension prevalence (P = 0.01), and reported a higher smoking rate (P = 0.01). No significant differences were observed for diabetes prevalence, marital status, alcohol consumption, or pregnancy history between the two groups.

**Table 1:** Baseline characteristics of study participants.

**Variables**	**Control** **n = 1903**	**Premature Menopause** **n = 662**	***P* value**
Age	63.59 (0.28)	53.56 (0.68)	<0.001
BMI	29.18 (0.23)	29.61 (0.35)	0.31
PIR	3.03 (0.06)	2.66 (0.08)	<0.001
1-NAP	55351.19 (13707.89)	164841.61 (83070.35)	0.20
2-NAP	5969.11 (286.66)	9164.15 (568.02)	<0.001
3-FLU	193.01 (14.35)	365.84 (35.32)	<0.001
2-FLU	404.27 (28.94)	684.20 (55.82)	<0.001
1-PHE	165.41 (7.24)	209.02 (15.82)	0.01
1-PYR	136.21 (11.25)	204.56 (23.56)	0.01
Urinary creatinine	83.99 (1.56)	101.55 (3.13)	<0.001
Race			0.03
White	924 (76.51)	321 (73.35)	
Mexican	272 (4.51)	90 (5.61)	
Black	370 (10.14)	172 (13.47)	
Other	337 (8.84)	79 (7.58)	
Education			0.001
Less High School	523 (17.76)	212 (24.74)	
High School	422 (21.96)	146 (24.39)	
Above High School	958 (60.28)	304 (50.87)	
Pregnancy			0.62
No	164 (9.97)	68 (10.83)	
Yes	1739 (90.03)	594 (89.17)	
Marital Status			0.24
Separated	814 (37.79)	253 (34.25)	
Married	934 (55.08)	333 (56.62)	
Never Married	155 (7.13)	76 (9.12)	
Diabetes			0.70
No	1552 (85.48)	544 (86.14)	
Yes	351 (14.52)	118 (13.86)	
Hypertension			0.01
No	838 (48.91)	341 (55.89)	
Yes	1065 (51.09)	321 (44.11)	
Smoking			0.01
No	1148 (56.38)	339(47.85)	
Yes	755 (43.62)	323(52.15)	
Alcohol using			0.25
No	456 (17.69)	129 (15.55)	
Yes	1447 (82.31)	533 (84.45)	

BMI, body mass index; PIR, poverty-to-income ratio; 1-NAP, 1-hydroxynaphthalene; 2-NAP, 2-hydroxynaphthalene; 3-FLU, 3-hydroxyfluorene; 2-FLU, 2-hydroxyfluorene; 1-PHE, 1-hydroxyphenanthrene; 1-PYR, 1-hydroxypyrene.

### Association between UPAHMs and premature menopause

Table 2 summarizes the associations between UPAHMs and premature menopause based on weighted multivariable regression models. In the unadjusted model (Model 1), higher levels of 1-NAP (OR: 1.01, 95% CI: 1.00–1.02, P = 0.01), 2-NAP (OR: 1.03, 95% CI: 1.02–1.04, P < 0.001), 3-FLU (OR: 1.06, 95% CI: 1.03–1.08, P < 0.001), 2-FLU (OR: 1.03, 95% CI: 1.01–1.04, P < 0.001), and 1-PHE (OR: 1.07, 95% CI: 1.01–1.12, P = 0.02) were significantly associated with premature menopause. These associations remained significant after adjusting for race, BMI, education, and PIR in Model 2 and were consistent after further adjustment for smoking and alcohol consumption in Model 3. In the fully adjusted Model 4, significant associations persisted for 1-NAP (OR: 1.01, 95% CI: 1.00–1.02, P = 0.02), 2-NAP (OR: 1.01, 95% CI: 1.00–1.02, P = 0.02), and 3-FLU (OR: 1.03, 95% CI: 1.01–1.05, P = 0.01), while associations for other metabolites were no longer significant. These findings indicate that elevated levels of specific PAH metabolites independently contribute to higher premature menopause risk.

**Table 2:** Association between UPAHMs and premature menopause.

**Exposures**	**OR (95% CI) *P* value**			

	**Model 1**	**Model 2**	**Model 3**	**Model 4**
1-NAP	1.01 (1.00,1.02) 0.01	1.01 (1.00,1.02) 0.01	1.01 (1.00,1.02) 0.02	1.01 (1.00,1.02) 0.02
2-NAP	1.03 (1.02,1.04) <0.001	1.03 (1.01,1.04) <0.001	1.02 (1.01,1.03) <0.001	1.01 (1.00,1.02) 0.02
3-FLU	1.06 (1.03,1.08) <0.001	1.05 (1.03,1.08) <0.001	1.05 (1.02,1.07) <0.001	1.03 (1.01,1.05) 0.01
2-FLU	1.03 (1.01,1.04) <0.001	1.03 (1.01,1.04) <0.001	1.02 (1.01,1.04) 0.003	1.01 (1.00,1.03) 0.050
1-PHE	1.07 (1.01,1.12) 0.02	1.06 (1.00,1.12) 0.04	1.05 (1.00,1.11) 0.049	1.01 (0.97,1.06) 0.61
1-PYR	1.05 (0.98,1.12) 0.13	1.04 (0.98,1.11) 0.16	1.04 (0.98,1.09) 0.20	1.01 (0.98,1.05) 0.49

Model 1: Non-adjusted.

Model 2: Adjusted for race, BMI, education and PIR.

Model 3: Further adjusted for Smoking and alcohol using based on model 2.

Model 4: Further adjusted for pregnancy, marital status, hypertension, diabetes and urinary creatinine based on model 3.

### Non-linear associations between UPAHMs and premature menopause

**Fig. 2:**
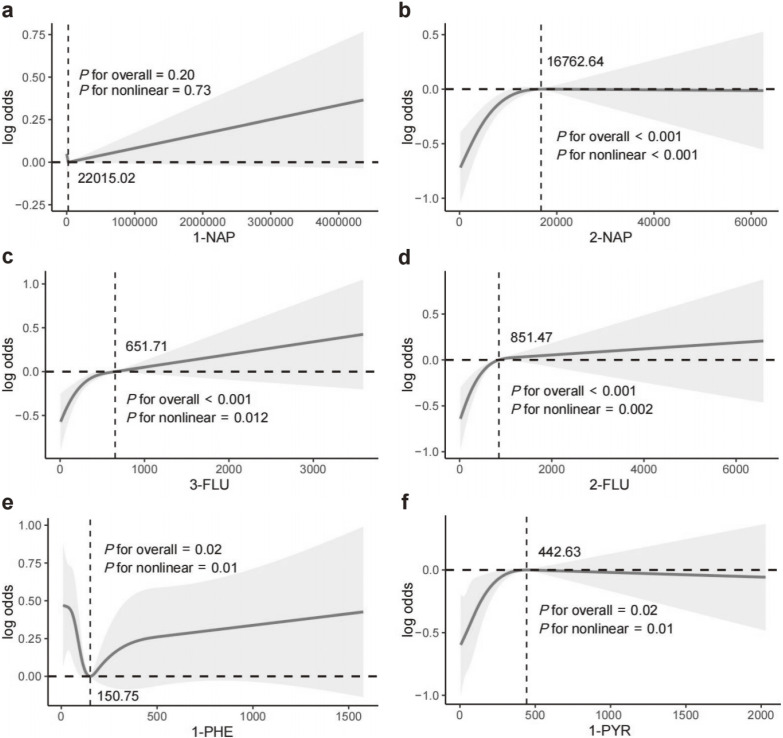
Restricted cubic spline analysis of the association between UPAHMs and premature menopause risk. Solid lines represent log odds ratios, shaded areas indicate 95% confidence intervals (CIs), and vertical dashed lines denote threshold concentrations. (a) 1-NAP showed no significant association, while (b) 2-NAP, (c) 3-FLU, (d) 2-FLU, and (f) 1-PYR exhibited nonlinear associations, and (e) 1-PHE demonstrated a U-shaped association.

RCS analyses (Fig. 2) revealed nonlinear relationships between UPAHMs and premature menopause risk. No significant association was found for 1-NAP (P for overall = 0.20, P for nonlinearity = 0.73). For 2-NAP, the risk increased sharply at lower concentrations and plateaued beyond 16,762.64 ng/mL (P for overall < 0.001, P for nonlinearity < 0.001). Similar patterns were observed for 3-FLU and 2-FLU, with risks stabilizing beyond 651.71 ng/mL and 851.47 ng/mL, respectively (P for overall < 0.001; P for nonlinearity = 0.01 for 3-FLU, P = 0.002 for 2-FLU).

For 1-PHE, a U-shaped relationship was identified, with risk decreasing at lower concentrations and rising after 150.75 ng/mL (P for overall = 0.02, P for nonlinearity = 0.01). Likewise, 1-PYR exhibited a sharp risk increase at lower concentrations, leveling off beyond 442.63 ng/mL (P for overall = 0.02, P for nonlinearity = 0.01). These findings suggest that dose-response relationships differ among PAH metabolites, with certain metabolites displaying threshold effects.

### Subgroup analysis

**Fig. 3:**
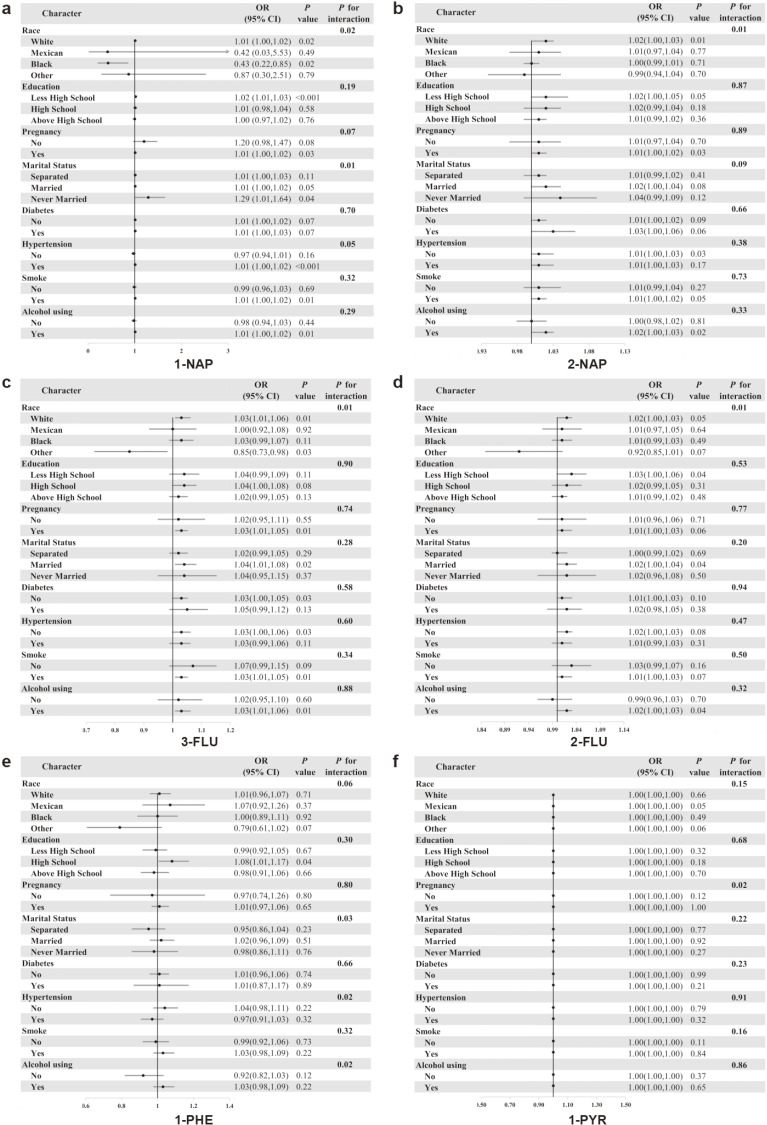
Subgroup analysis of the association between UPAHMs and premature menopause risk. Forest plots display odds ratios (ORs) with 95% CIs for the associations between six UPAHMs and premature menopause across different subgroups. (a) 1-NAP, (b) 2-NAP, (c) 3-FLU, (d) 2-FLU, (e) 1-PHE, and (f) 1-PYR.

Subgroup analysis results (Fig. 3) demonstrated heterogeneity in the association between UPAHMs and premature menopause risk across demographic and clinical characteristics. Race significantly modified these associations. For 1-NAP, a protective effect was observed among Black participants, while increased risks were noted among White participants (P for interaction = 0.02). Similarly, 2-NAP, 3-FLU, and 2-FLU showed significant positive associations only among White participants (P for interaction = 0.01 for all).

Marital status and hypertension influenced the relationship between 1-NAP and premature menopause, with positive associations observed among unmarried individuals and those with hypertension (P for interaction = 0.01 and P < 0.05, respectively). For 1-PHE, significant interactions were noted with marital status (P = 0.03), hypertension (P = 0.02), and alcohol consumption (P = 0.02). Additionally, pregnancy history significantly modified the association between 1-PYR and premature menopause (P for interaction = 0.02). These results highlight the importance of demographic and clinical factors in modulating the impact of PAH metabolites on premature menopause risk.

## Discussion

This study, utilizing nationally representative data from the 2001–2020 NHANES, comprehensively evaluated the association between UPAHMs and premature menopause risk. Elevated levels of 1-NAP, 2-NAP, and 3-FLU were significantly linked to higher risk, with RCS analysis revealing nonlinear relationships. Subgroup analyses highlighted race as a critical modifier, with White women showing higher susceptibility. These findings underscore the impact of environmental pollutants on ovarian function and provide a foundation for targeted interventions.

Our findings align with experimental and epidemiological research on the complex mechanisms of PAH-induced ovarian toxicity [28–32]. PAHs are metabolized by cytochrome P450 enzymes, primarily CYP1A1 and CYP1B1, into reactive intermediates such as epoxides and dihydrodiols [33]. These reactive metabolites generate oxidative stress, induce DNA damage, and impair mitochondrial function in granulosa cells, accelerating follicular atresia and depleting ovarian reserves [15]. Moreover, PAHs activate the aryl hydrocarbon receptor (AhR) pathway, disrupting the hypothalamic-pituitary-ovarian axis. This disruption alters steroidogenesis and hormonal homeostasis, further impairing ovarian function [34–36]. Oxidative stress plays a pivotal role in granulosa cell apoptosis and oocyte damage [2]. For instance, benzo[a]pyrene has been shown to increase reactive oxygen species (ROS) levels, reduce antioxidant enzyme activity, and compromise follicular integrity and organelle function [17, 34, 37]. PAHs’ endocrine-disrupting properties add another layer of complexity [38], as they interact with estrogen receptors and downregulate aromatase expression, reducing estradiol synthesis [39]. This hormonal imbalance adversely affects follicular growth, ovulation, and overall reproductive capacity [40]. Our findings further support these mechanisms, showing that exposure to metabolites such as 1-NAP, 2-NAP, and 3-FLU is significantly associated with accelerated ovarian aging and an increased risk of premature menopause.

Through RCS analysis, we were the first to identify a nonlinear relationship between UPAHMs levels and the risk of premature menopause in a large population-based cohort. For 2-NAP, 3-FLU, 2-FLU, and 1-PYR, the risk of premature menopause increased sharply at lower exposure levels but plateaued at higher concentrations. This pattern may be attributed to the rapid cumulative damage induced by oxidative stress and inflammation in granulosa cells and follicular reserves at low exposure levels, whereas at higher exposure levels, ovarian function may reach a threshold of maximal damage, leading to a saturation effect [41, 42]. Additionally, the U-shaped relationship observed for 1-PHE suggests heterogeneous effects of PAH metabolites on ovarian function. Lower concentrations of 1-PHE may confer a protective effect by activating antioxidant enzymes or DNA repair mechanisms; however, at higher concentrations, the risk increases significantly due to excessive oxidative stress and disruption of the ovarian microenvironment. These findings underscore the diverse impacts of PAH metabolites, which are influenced by their chemical properties and metabolic pathways, providing a basis for developing targeted public health interventions for individuals with high exposure and an elevated risk of premature menopause.

Subgroup analysis revealed significant racial disparities in the association between UPAHMs and premature menopause risk. For 1-NAP and 3-FLU, these metabolites exhibited a protective effect among Black participants but were associated with an increased risk among White participants. Additionally, 2-NAP and 2-FLU were positively associated with premature menopause risk exclusively in White participants. One plausible explanation for these disparities is genetic polymorphisms in metabolic enzymes, such as CYP1A1 and CYP1B1, which metabolize PAHs into reactive intermediates [43–45]. These polymorphisms vary across racial groups, influencing PAH metabolism efficiency and toxicity. Such genetic differences may explain why White participants demonstrated greater sensitivity to specific PAH metabolites, whereas Black participants exhibited a more attenuated response. Environmental and lifestyle factors may further amplify these disparities. Socioeconomic differences can influence PAH exposure levels, as individuals residing or working in industrialized or urban areas are more likely to experience elevated occupational or environmental exposure [46, 47]. These findings highlight the need for targeted public health interventions to reduce PAH exposure, particularly among high-risk populations such as White individuals, who appear to be more vulnerable to the reproductive effects of PAH metabolites.

This study has several strengths. First, we utilized NHANES data, which is nationally representative and undergoes rigorous quality control, enhancing the generalizability of our findings. Second, we employed robust statistical methods to evaluate both linear and nonlinear relationships between PAH exposure and premature menopause risk. However, the study has limitations. First, its cross-sectional design prevents causal inference, necessitating longitudinal validation. Future studies should incorporate prospective cohorts to establish temporal relationships. Additionally, mechanistic studies exploring how PAHs disrupt ovarian function could provide further biological plausibility. Second, urinary PAH metabolites reflect only short-term exposure, which may not capture long-term cumulative effects on ovarian function. Future research should consider repeated biomarker measurements or alternative indicators of chronic exposure to enhance exposure assessment. Third, while we adjusted for multiple confounders, unmeasured lifestyle factors, such as dietary intake, occupational exposure, and co-exposure to other endocrine-disrupting chemicals, could still influence the results. Lastly, the sample size in subgroup analyses may limit statistical power, requiring validation in larger cohorts.

In the U.S., policies such as the Clean Air Act regulate atmospheric PAH emissions. However, our findings indicate that existing measures may require further reinforcement to fully mitigate exposure risks, particularly among high-risk groups. Enhanced emission controls, expanded air quality monitoring, and stricter workplace protections for PAH-exposed occupations are critical to reducing health risks. Despite its limitations, this study provides strong epidemiological evidence linking UPAHMs to premature menopause in a nationally representative population. Notably, White women exhibited greater susceptibility, highlighting the need for individualized risk assessment. Clinically, environmental pollutants should be incorporated into premature menopause risk evaluations, particularly in vulnerable groups. Public health strategies should prioritize reducing PAH exposure through tighter regulatory oversight, occupational safety measures, and targeted smoking cessation programs. Strengthened policies remain essential to minimizing PAH exposure and safeguarding women’s reproductive health.

## Conclusions

This study identified significant associations between elevated UPAHMs levels and an increased risk of premature menopause, with evidence of nonlinear relationships. White women exhibited greater sensitivity to PAH exposure, highlighting the importance of considering individual characteristics in risk assessment. These findings underscore the need for enhanced public health policies and strengthened regulatory measures to limit PAH exposure, particularly among high-risk populations. Reducing environmental PAH exposure is crucial for protecting women’s reproductive health and preserving ovarian function.

## Abbreviations

PAHs: Polycyclic Aromatic Hydrocarbons
UPAHMs: Urinary Polycyclic Aromatic Hydrocarbon Metabolites
EPA: Environmental Protection Agency
NHANES: National Health and Nutrition Examination Survey
POI: Premature Ovarian Insufficiency
RCS: Restricted Cubic Spline
CYP: Cytochrome P450
1-NAP: 1-Hydroxynaphthalene
2-NAP: 2-Hydroxynaphthalene
3-FLU: 3-Hydroxyfluorene
2-FLU: 2-Hydroxyfluorene
1-PHE: 1-Hydroxyphenanthrene
1-PYR: 1-Hydroxypyrene
ROS: Reactive Oxygen Species
PIR: Poverty-to-Income Ratio
OR: Odds Ratio
CI: Confidence Interval
AhR: Aryl Hydrocarbon Receptor

## Declarations

### Each author’s participation in the manuscript

QY and LLZ contributed equally to this work and are joint first authors. QY contributed to Conceptualization, Methodology, Formal Analysis, Data Curation, and Writing – Original Draft Preparation. LLZ contributed to Investigation, Data Collection, Methodology, and Writing – Review & Editing. JFH contributed to Visualization and Formal Analysis. JXW contributed to Data Curation and Validation. HL contributed to Investigation and Validation. KXD, as the corresponding author, contributed to Supervision, Project Administration, Funding Acquisition, and Writing – Review & Editing. All authors have read and approved the final manuscript.

### Availability of data and materials

The datasets used and analyzed during the current study are available from publicly accessible repositories. Data for this study were obtained from the NHANES database, which is publicly available at https://www.cdc.gov/nchs/nhanes/index.htm. Further details or materials can be requested from the corresponding author upon reasonable request.
